# The effect of serum lipase levels on clinical severity scores and mortality in acute pancreatitis: a retrospective cohort study

**DOI:** 10.1186/s12876-026-04897-0

**Published:** 2026-05-01

**Authors:** Mustafa Batuhan Kilicaslan, Ozlem Dikme, Mehmet Can Girgin, Erdem Kurt, Ozgür Dikme

**Affiliations:** Department of Emergency Medicine, SBU Istanbul Training and Research Hospital, Istanbul, Turkey

**Keywords:** Acute pancreatitis, Serum lipase, Prognostic score, Mortality

## Abstract

**Aims:**

This study aimed to investigate the relationship between admission serum lipase levels, prognostic clinical scoring systems, and in-hospital mortality in patients presenting to the emergency department with acute pancreatitis.

**Methods:**

This retrospective cohort study included 389 patients hospitalized with acute pancreatitis at a tertiary care center between 2021 and 2023. Demographic characteristics, clinical findings, laboratory parameters obtained at admission and at 48 h, and imaging findings were collected. Prognostic assessment was performed using the Ranson, APACHE II, BISAP, Glasgow–Imrie, HAPS, and CTSI scoring systems. Patients were categorized according to a predefined serum lipase cutoff of 600 U/L. The associations between serum lipase levels, prognostic score categories, and in-hospital mortality were analyzed, and the diagnostic performance of the lipase cutoff was evaluated.

**Results:**

The overall in-hospital mortality rate was 3.9% (*n* = 15). Admission serum lipase levels were significantly associated with the Ranson score (*p* < 0.05) and several laboratory parameters. A serum lipase cutoff of 600 U/L identified patients with a Ranson score ≥ 3 with a sensitivity of 70.2% (95% CI: 60.4–78.8) and a specificity of 57.3% (95% CI: 51.2–63.2). Higher prognostic scores, including Ranson, BISAP, and APACHE II, were significantly associated with in-hospital mortality (*p* < 0.01). However, admission serum lipase levels were not significantly associated with in-hospital mortality.

**Conclusion:**

Admission serum lipase showed a limited association with selected prognostic indicators in acute pancreatitis but was not associated with in-hospital mortality. These findings suggest that serum lipase has limited value as a standalone prognostic marker and should be interpreted in conjunction with established clinical scoring systems. Further prospective studies are needed to better clarify its role in risk assessment.

## Introduction

Acute pancreatitis (AP) is an inflammatory condition of the pancreas caused by the premature activation of pancreatic digestive enzymes. Its clinical course can vary widely, ranging from a mild and self-limiting illness to a severe, potentially life-threatening disease. Gallstone disease and excessive alcohol consumption are the most common etiological factors and are both well-established causes of pancreatic injury and inflammation [1]. The diagnosis of AP is based on the presence of at least two of the following three criteria: characteristic abdominal pain, elevated serum pancreatic enzyme levels, and imaging findings consistent with acute pancreatitis [2]. Approximately 75–85% of acute pancreatitis cases are mild. Advances in supportive care and early management strategies have been associated with a reduction in mortality rates to approximately 1–2% in recent studies [3].

Despite these improvements, early identification of patients at increased risk of poor clinical outcomes remains essential for timely intervention and the prevention of complications. Several clinical scoring systems, including Ranson, BISAP, and APACHE II, are widely used for prognostic assessment and risk stratification in acute pancreatitis. In addition, imaging-based scoring systems such as the Balthazar classification and the Computed Tomography Severity Index (CTSI) provide useful information regarding pancreatic inflammation and necrosis [4].

Serum lipase is a key biomarker in the diagnosis of acute pancreatitis and is generally preferred over amylase because of its higher sensitivity and specificity, as well as its longer duration of elevation, which is particularly useful in patients presenting later after symptom onset [5]. However, the prognostic value of serum lipase remains controversial. Although established clinical scoring systems are widely used, they also have important limitations. For example, the Ranson criteria require the assessment of 11 parameters over a 48-hour period, which limits their utility during the early phase of disease and in high-turnover settings such as emergency departments [6]. Similarly, although imaging-based indices such as the CTSI can provide additional prognostic information, their routine use may be limited by concerns related to standardization, radiation exposure, and availability across different healthcare settings [7].

Given these limitations, there is a need for simple and readily available markers that may complement existing scoring systems and support early risk stratification. Therefore, this study aimed to evaluate the association between serum lipase levels, prognostic clinical scores, and in-hospital mortality in patients with acute pancreatitis. However, current guideline-level evidence primarily supports serum lipase as a diagnostic marker rather than an established prognostic marker in acute pancreatitis.

## Materials and methods

This retrospective cohort study was conducted in the emergency department of SBU Istanbul Training and Research Hospital, a tertiary care center, between January 2021 and December 2023. The study was designed to evaluate the association between serum lipase levels, prognostic clinical scores, and in-hospital outcomes in patients diagnosed with acute pancreatitis (AP). The study protocol was approved by the institution’s Clinical Research Ethics Committee (approval date: 15.09.2023; decision no: 231). Data collection was initiated after ethical approval and was based exclusively on routinely obtained diagnostic, therapeutic, and follow-up data, without any additional interventions.

Eligible participants consisted of consecutive patients aged 18 years or older who were hospitalized with a confirmed diagnosis of AP. The diagnosis was established based on the presence of at least two of the following three criteria: characteristic abdominal pain, serum lipase or amylase levels at least three times the upper limit of normal (ULN), defined according to the reference range used at our institution (0–67 U/L for serum lipase), or imaging findings consistent with acute pancreatitis. Inclusion required the availability of serum lipase measurements at admission. Exclusion criteria included chronic pancreatitis, pancreatic malignancy, cardiac or respiratory arrest at initial presentation, trauma-related pancreatitis, pregnancy, and incomplete medical records. Patient identification was performed through the hospital’s electronic medical record system, and no sampling method was applied; all eligible patients during the study period were included.

The serum lipase cutoff value of 600 U/L was predefined a priori based on previous literature evaluating its relationship with disease severity and radiologic indices, rather than being derived from the present dataset [8]. This cutoff corresponds to approximately 9× ULN in our laboratory.

### Variables and clinical scoring systems

The primary outcome of the study was in-hospital mortality. Secondary outcomes included length of hospital stay and intensive care unit admission. Predictor variables included serum lipase levels and prognostic clinical scores, specifically the Ranson, BISAP, APACHE II, Glasgow–Imrie, HAPS, and CTSI scores. Potential confounders included age, sex, comorbidities, and vital signs at presentation.

The Ranson score was calculated using 11 clinical and laboratory parameters assessed at admission and at 48 h, with a score of ≥ 3 indicating a higher-risk category [3]. The BISAP score was calculated using five parameters, including blood urea nitrogen level and the presence of systemic inflammatory response syndrome, with a score of ≥ 3 indicating a higher-risk category [9]. The APACHE II score, which incorporates physiological variables and laboratory findings, was calculated at admission, and a score of ≥ 8 was used to define a higher-risk category [10]. Abdominal CT findings were assessed using contrast-enhanced abdominal CT scans obtained within 48–72 h of hospital admission, together with the corresponding radiology reports issued by the radiology department. The CTSI score was calculated based on the evaluation of pancreatic inflammation, pancreatic parenchymal edema, peripancreatic fluid collections, and pancreatic necrosis. Radiologic severity was graded using the CTSI, which incorporates the Balthazar classification and the extent of pancreatic necrosis [8]. The Glasgow–Imrie score was calculated using standard biochemical and clinical parameters obtained within the first 48 h of hospitalization, and a score of ≥ 3 was considered a higher-risk category [11]. The Harmless Acute Pancreatitis Score (HAPS) was calculated using standard admission parameters and categorized according to established criteria [11].

### Data collection and quality control

Data were extracted from the hospital information management system by two independent researchers who completed a one-day standardization training to ensure consistent data collection. Inter-rater reliability was assessed for selected categorical variables using Cohen’s kappa coefficient, which demonstrated high agreement (κ = 0.85), thereby reducing the risk of information bias. Collected variables included demographic characteristics (age and sex), clinical parameters (vital signs and Glasgow Coma Scale score), laboratory findings (complete blood count, biochemical parameters, and serum lipase levels), imaging findings (computed tomography results), and clinical outcomes (mortality and length of hospital stay). Higher-risk comorbidities were operationally defined as liver cirrhosis, chronic kidney disease, heart failure, or immunosuppressive conditions. Clinical and laboratory parameters were primarily recorded at admission. However, variables required for score-based assessments (e.g., Ranson criteria) and selected laboratory parameters were also collected at 48 h after admission, as reflected in the relevant tables.

Serum lipase levels were measured using automated enzymatic assays according to routine institutional laboratory procedures. To minimize selection bias, all eligible patients were consecutively included.

### Sample size and statistical considerations

In acute pancreatitis, the estimated incidence of more severe disease ranges between 15% and 25%. Although the overall mortality rate among all AP cases is relatively low, it may increase substantially in patients with more severe clinical courses [3]. Based on these estimates, a power analysis was performed. Assuming a two-sided alpha error of 5%, a 95% confidence level, and a moderate effect size for comparisons between two independent groups, the minimum required sample size was calculated as 70 patients in the higher-risk group and 280 patients in the lower-risk group [4].

**Fig. 1:**
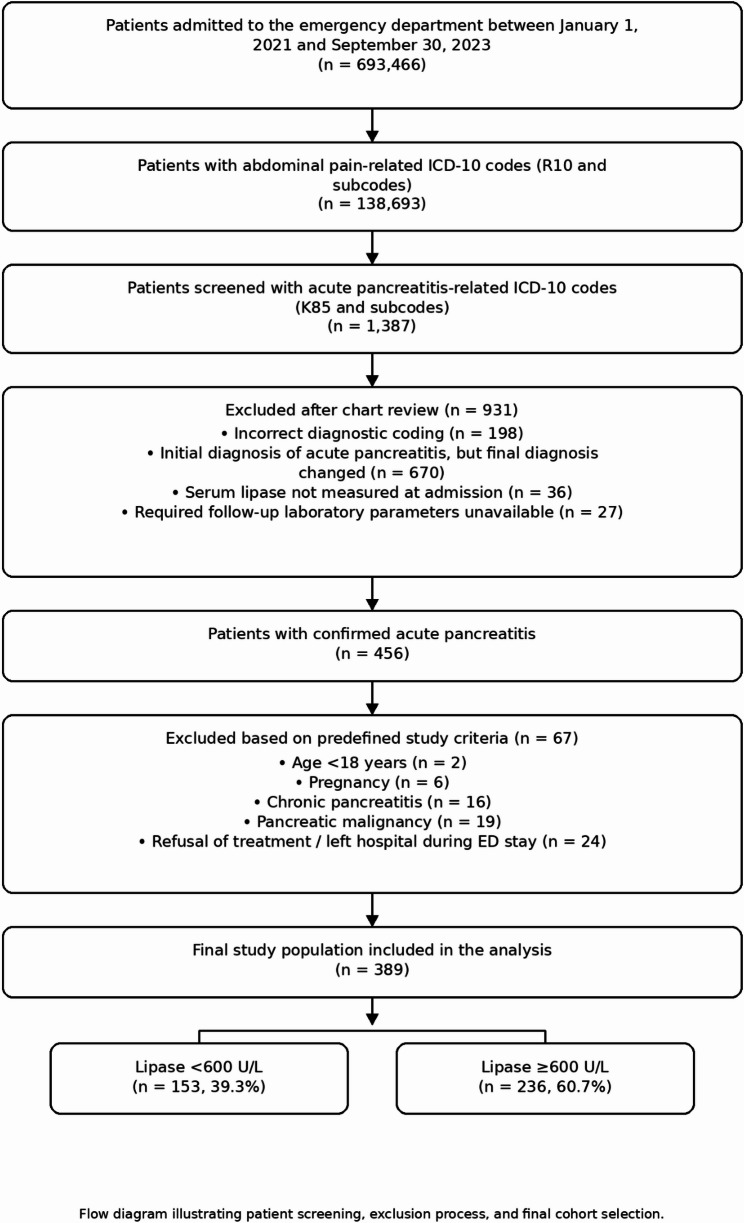
Flow diagram of patient selection

Continuous variables, such as serum lipase levels and age, were analyzed in both continuous and categorical forms. Categorical variables, including mortality and the presence of comorbidities, were analyzed as binary or ordinal variables, as appropriate. Patients who did not fulfill the inclusion criteria or met any of the exclusion criteria were excluded from the study, as illustrated in Fig. 1.

### Statistical analysis

Statistical analyses were performed using IBM SPSS Statistics version 28.0 (IBM Corp., Armonk, NY, USA). Continuous variables were assessed for normality using the Kolmogorov–Smirnov test together with visual inspection of histograms. Normally distributed variables were summarized as mean ± standard deviation and compared using the independent samples t-test, whereas non-normally distributed variables were summarized as median with interquartile range and compared using the Mann–Whitney U test.

Categorical variables were presented as frequencies and percentages and compared using the chi-square test or Fisher’s exact test, as appropriate. Receiver operating characteristic (ROC) curve analysis was performed to evaluate the discriminatory performance of serum lipase in identifying higher-risk score categories, and discriminatory ability was quantified by calculating the area under the curve (AUC).

To identify independent predictors of in-hospital mortality, multivariable logistic regression analysis was performed, and the results were reported as odds ratios with 95% confidence intervals. A two-sided p-value of < 0.05 was considered statistically significant.

## Results

### Participants

A total of 389 patients diagnosed with acute pancreatitis (AP) who presented to the emergency department of SBU Istanbul Training and Research Hospital between January 2021 and December 2023 were included in the analysis. The mean age of the study population was 56.0 ± 17.8 years, and 208 patients (53.5%) were male. At least one comorbid condition was present in 223 patients (57.3%), while 26 patients (6.7%) had higher-risk comorbidities, including liver cirrhosis, chronic kidney disease, heart failure, or immunosuppressive conditions. The overall in-hospital mortality rate was 3.9% (*n* = 15).

The baseline categorical characteristics of the study population are presented in Table 1.

**Table 1:** Categorical characteristics and distributions of the patients included in the study

Characteristic	*n* (389)	%
Gender		
Male	208	53.5
Female	181	46.5
Comorbidities		
Present	223	57.3
Absent	166	42.7
Higher-risk Comorbidities^a^		
Present	26	6.7
Absent	363	93.3
Abdominal Examination		
Positive	258	66.3
Negative	131	33.7
Abdominal CT Findings		
Normal	143	36.8
1–4 Pancreatitis	240	61.7
≥ 4 Necrosis	6	1.5
Emergency Department Outcome		
Ward Admission	369	94.9
ICU Admission	20	5.1
Surgical Requirement		
Present	13	3.3
Absent	376	96.7
Pleural Effusion (24 h)		
Present	33	8.5
Absent	356	91.5
Mortality		
Present	15	3.9
Absent	374	96.1

^a^Higher-risk comorbidities include liver cirrhosis, chronic kidney disease, heart failure, or immunosuppression

The numerical characteristics and distributions of the patients included in the study are presented in Table 2.

**Table 2:** Numerical characteristics and distributions of the patients included in the study

Parameter	Min.	Max.	Median	Mean ± SD
Age	18	106	56	56 ± 17.8
Mean Arterial Pressure	58	140	91	92.3 ± 12.5
Heart Rate	50	186	78	80.5 ± 15.1
Temperature	35	39.3	36.5	36.6 ± 0.5
Respiratory Rate	12	28	14	14.4 ± 1.6
GCS	10	15	15	15 ± 0.4
Fluid Intake (L)	0	10	4.5	4.5 ± 1.7
Hospital Stay (day)	1	60	5	6.3 ± 5.2

*SD* Standard Deviation, *GCS* Glasgow Coma Scale

Clinical parameters and severity scoring systems according to serum lipase levels are summarized in Table 3.

**Table 3:** Clinical characteristics, outcomes, and scoring systems by serum lipase levels

Parameter	Overall (*N* = 389)	Lipase < 600 U/L (*n* = 153)	Lipase ≥ 600 U/L (*n* = 236)	*p* Value
Clinical Parameters (Mean ± SD)				
Age	56 ± 17.8	54.3 ± 16.5	57.8 ± 18.2	0.042
AST (IU/L)	137.3 ± 218.2	70.4 ± 121.6	180.7 ± 253.5	< 0.001
LDH (IU/L)	313.1 ± 160.4	284.8 ± 142.3	331.5 ± 168.9	0.004
pH	7.38 ± 0.05	7.39 ± 0.05	7.38 ± 0.04	0.036
pCO₂ (mmHg)	43.0 ± 5.0	42.2 ± 5.4	43.6 ± 4.6	0.009
Clinical Scores (Mean ± SD)				
Ranson Score	1.8 ± 1.4	1.6 ± 1.3	2.0 ± 1.5	0.005
Glasgow Score	1.5 ± 1.3	1.4 ± 1.2	1.5 ± 1.4	0.355
APACHE II Score	4.9 ± 3.7	4.7 ± 3.5	5.0 ± 3.8	0.442
BISAP Score	0.8 ± 0.9	0.75 ± 0.89	0.81 ± 0.97	0.512
HAPS Score	1.1 ± 0.7	1.05 ± 0.72	1.09 ± 0.70	0.621
CTSI Score	1.5 ± 1.5	1.51 ± 1.51	1.42 ± 1.46	0.560
Categorical Evaluation, n (%)				
Ranson Score < 3	295 (75.8)	125 (81.7)	170 (72.0)	0.019
Ranson Score ≥ 3	94 (24.2)	28 (18.3)	66 (28.0)	
APACHE II Score < 8	310 (79.7)	120 (78.4)	190 (80.5)	0.355
APACHE II Score ≥ 8	79 (20.3)	33 (21.6)	46 (19.5)	
BISAP Score < 3	370 (95.1)	147 (96.1)	223 (94.5)	0.325
BISAP Score ≥ 3	19 (4.9)	6 (3.9)	13 (5.5)	
Glasgow Score < 3	315 (81.0)	128 (83.7)	187 (79.2)	0.170
Glasgow Score ≥ 3	74 (19.0)	25 (16.3)	49 (20.8)	
HAPS Score < 1	83 (21.3)	36 (23.5)	47 (19.9)	0.234
HAPS Score ≥ 1	306 (78.7)	117 (76.5)	189 (80.1)	
CTSI Score < 4	363 (93.3)	142 (92.8)	221 (93.6)	0.450
CTSI Score ≥ 4	26 (6.7)	11 (7.2)	15 (6.4)	
Mortality (+)	15(3.9)	6 (3.9)	9 (3.8)	
Mortality (-)	374(96.1)	147 (96.1)	227 (96.2)	0.957

Continuous variables are presented as mean ± standard deviation. Group comparisons were performed using the independent samples t-test or Mann–Whitney U test, depending on data distribution. Categorical variables are presented as number (percentage) and were compared using the chi-square test or Fisher’s exact test, as appropriate. Unless otherwise specified, variables represent admission values.

The relationship between clinical score categories and mortality is shown in Table 4.

**Table 4:** Relationship Between Clinical Score Categories and Mortality

Variable	Mortality (+) (*N* = 15)	Mortality (-) (*N* = 374)	*P* Value
	*n* (%)	*n* (%)	
Ranson Score			0.007
<3	7 (46.7)	288 (77)	
≥3	8 (53.3)	86 (23)	
Glasgow Score			0.442
<3	11 (73.3)	304 (81.3)	
≥3	4 (26.7)	70 (18.7)	
APACHE II Score			< 0.001
<8	3 (20.0)	307 (82.1)	
≥8	12 (80.0)	67 (17.9)	
BISAP Score			< 0.001
<3	10 (66.7)	360 (96.3)	
≥3	5 (33.3)	14 (3.7)	
HAPS Score			0.607
<1	4 (26.7)	79 (21.1)	
≥1	11 (73.3)	295 (78.9)	
CTSI Score			1.000
<4	14 (93.3)	349 (93.3)	
≥4	1 (6.7)	25 (6.7)	

The relationship between laboratory parameters and in-hospital mortality is presented in Table 5.

**Table 5:** Relationship Between Laboratory Parameters and In-Hospital Mortality

Parameter	Mortality (+) (*N* = 15)	Mortality (-) (*N* = 374)	*p* Value
	Mean ± SD	Mean ± SD	
Admission Values			
WBC(×10³ / µL)	11.9 ± 5.2	9.1 ± 3.3	0.015
HCT(%)	36.8 ± 8.0	39.8 ± 5.6	0.046
Glucose(mg/dL)	147.6 ± 65.7	119.2 ± 37.7	0.117
Urea(mg/dL)	61.8 ± 68.7	36.3 ± 21.3	0.049
Creatinine(mg/dL)	1.4 ± 1.1	0.9 ± 0.6	0.235
Sodium(mEq/L)	138.0 ± 4.8	140.0 ± 3.9	0.099
Potassium(mEq/L)	4.2 ± 0.6	4.3 ± 0.8	0.739
AST(IU/L)	139.0 ± 221.0	96.0 ± 129.2	0.749
LDH(IU/L)	448.4 ± 356.6	307.7 ± 145.7	0.100
pH	7.33 ± 0.12	7.38 ± 0.04	0.028
pCO2(mmHg)	41.1 ± 9.8	43.1 ± 4.7	0.935
Lipase(U/L)	1187.8 ± 1075.1	1561.1 ± 1534.7	0.632
48th Hour Values			
HCT(%)	34.2 ± 5.5	35.6 ± 5.8	0.359
Urea(mg/dL)	69.6 ± 62.7	26.2 ± 17.7	< 0.001
Albumin(g/L)	32.1 ± 8.0	36.3 ± 5.1	0.071
Calcium(mg/dL)	8.0 ± 1.0	8.6 ± 0.7	0.003
PaO2(mmHg)	73.1 ± 10.4	79.8 ± 2.2	< 0.001
pCO2(mmHg)	41.1 ± 10.0	43.1 ± 4.6	0.889
HCO3(mmol/L)	22.5 ± 6.4	25.7 ± 2.6	0.017
Base Excess(mmol/L)	-4.1 ± 7.1	-0.5 ± 2.8	0.104

Continuous variables are presented as mean ± standard deviation. Group comparisons were performed using the independent samples t-test or Mann–Whitney U test, as appropriate based on data distribution. Unless otherwise specified, variables represent admission values.

The diagnostic performance of serum lipase in identifying higher-risk score categories, as evaluated by ROC analysis, is presented in Table 6.

**Table 6:** ROC Analysis of Serum Lipase for Identifying Higher-Risk Clinical Score Categories

Severity Score	AUC	Standard Error	95% CI Lower	95% CI Upper	*p* Value
Ranson ≥ 3	0.585	0.034	0.519	0.651	0.013
Glasgow ≥ 3	0.565	0.038	0.491	0.639	0.083
APACHE II ≥ 8	0.504	0.040	0.425	0.583	0.911
BISAP ≥ 3	0.542	0.078	0.389	0.694	0.540
HAPS ≥ 1	0.563	0.036	0.493	0.633	0.078
CTSI ≥ 4	0.450	0.068	0.316	0.584	0.391

*AUC* Area Under the Curve, *CI* Confidence Interval, *ROC* Receiver Operating Characteristic

### Main results

Serum lipase levels, using a cutoff value of 600 U/L, were significantly associated with higher-risk Ranson score categories (*p* = 0.005). Patients with serum lipase levels ≥ 600 U/L had higher mean Ranson scores than those with levels < 600 U/L (2.0 ± 1.5 vs. 1.6 ± 1.3). Among patients with a Ranson score ≥ 3, 28.0% had serum lipase levels ≥ 600 U/L, whereas 18.3% had levels < 600 U/L (*p* = 0.019).

The diagnostic performance of a serum lipase cutoff of 600 U/L for identifying patients with Ranson ≥ 3 showed a sensitivity of 70.2% (95% CI: 60.4–78.8) and a specificity of 57.3% (95% CI: 51.2–63.2). Receiver operating characteristic (ROC) curve analysis yielded an area under the curve (AUC) of 0.585 (95% CI: 0.519–0.651; *p* = 0.013), indicating statistically significant but limited discriminative performance.

No significant association was observed between serum lipase levels and in-hospital mortality when patients were compared according to the predefined serum lipase cutoff of 600 U/L (*p* = 0.957) (Table 3). Mortality rates were similar between patients with serum lipase levels < 600 U/L and those with levels ≥ 600 U/L (3.9% vs. 3.8%, respectively). In addition, admission serum lipase levels, when evaluated as a continuous laboratory variable, did not differ significantly between patients who died and those who survived (*p* = 0.632) (Table 5).

Clinical scoring systems, including Ranson, APACHE II, and BISAP, were significantly associated with in-hospital mortality in univariate analyses (*p* = 0.007, *p* < 0.001, and *p* < 0.001, respectively). Mortality was higher among patients with a Ranson score ≥ 3 compared with those with scores < 3 (Table 4). Similarly, APACHE II scores ≥ 8 and BISAP scores ≥ 3 were associated with higher mortality rates. In contrast, Glasgow–Imrie, HAPS, and CTSI scores were not significantly associated with mortality (*p* = 0.442, *p* = 0.607, and *p* = 1.000, respectively).

Several laboratory parameters were significantly associated with in-hospital mortality. Patients who died had higher white blood cell counts and urea levels, as well as lower arterial pH, bicarbonate, hematocrit, and serum calcium levels (Table 5).

### Multivariable analysis

A multivariable logistic regression analysis was performed to evaluate independent predictors of in-hospital mortality. The model included the Ranson score and log-transformed serum lipase level. The Ranson score was independently associated with in-hospital mortality (OR: 1.52, 95% CI: 1.13–2.04, *p* = 0.006), whereas log-transformed serum lipase was not independently associated with mortality (OR: 0.90, 95% CI: 0.63–1.28, *p* = 0.546) (Table 7).

**Table 7:** Multivariable logistic regression analysis for in-hospital mortality

Variable	OR (Exp[B])	95% CI	*p*-value
Ranson score	1.52	1.13–2.04	0.006
Log-transformed lipase	0.90	0.63–1.28	0.546

*OR* odds ratio, *CI* confidence interval

### Other analyses

**Fig. 2:**
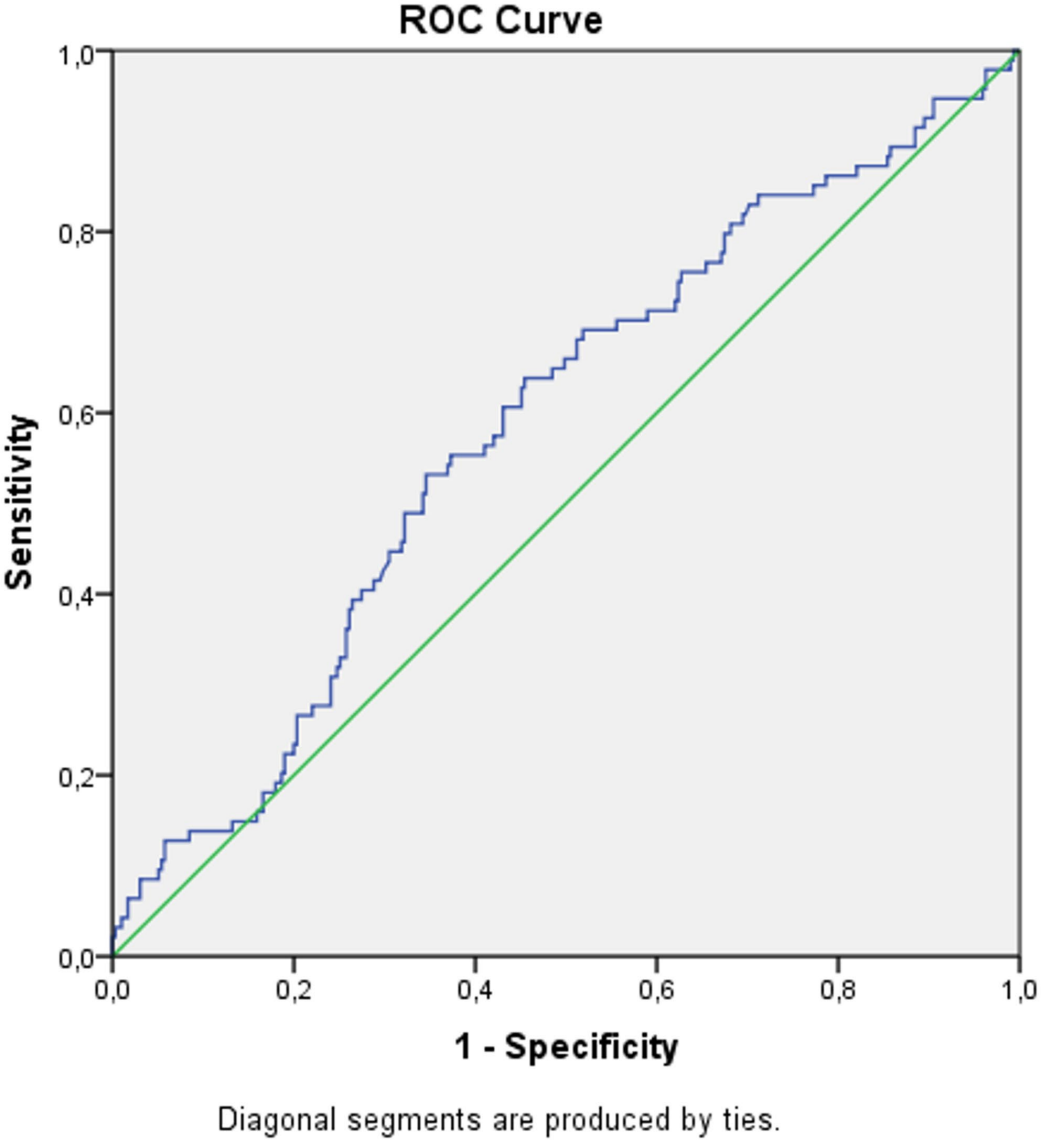
ROC Analysis of Lipase Level in Predicting the Ranson Score

Additional ROC analyses showed that the predictive performance of serum lipase for other clinical scoring systems was generally limited and not statistically significant (Table 6). The ROC curve analysis of serum lipase for identifying patients with Ranson ≥ 3 is shown in Fig. 2.

## Discussion

In this study, serum lipase levels, using a cutoff value of 600 U/L, showed a statistically significant but limited association with clinical risk stratification, particularly as reflected by the Ranson score (AUC: 0.585, 95% CI: 0.519–0.651; *p* = 0.013). However, serum lipase levels were not associated with in-hospital mortality, indicating limited prognostic value for fatal outcomes. These findings suggest that although serum lipase may be associated with selected risk indicators, it should not be relied upon as an independent predictor of mortality in acute pancreatitis.

Acute pancreatitis (AP) remains a clinically important condition because of its wide spectrum of presentation, ranging from mild, self-limiting inflammation to severe disease complicated by systemic inflammatory response and multiorgan failure. Epidemiological studies have consistently reported a higher prevalence of AP among male patients, although disease severity and outcomes may vary across populations [12, 13]. Previous studies have shown that etiological factors, particularly biliary pancreatitis, may influence serum lipase levels, suggesting that biomarker profiles can vary according to the underlying cause [14]. Despite advances in supportive care, mortality associated with acute pancreatitis remains a challenge, with reported in-hospital mortality rates ranging from 1% to 3% and higher mortality observed during post-discharge follow-up [15]. In addition, an average hospital stay of 4–6 days highlights the substantial healthcare burden associated with acute pancreatitis [16]. In this context, identifying simple and reliable prognostic markers remains an important clinical objective. However, current guideline-level evidence primarily supports serum lipase as a diagnostic marker rather than an established prognostic marker in acute pancreatitis.

The present study demonstrated a significant association between serum lipase levels and higher-risk Ranson score categories, but not with mortality, a finding that is consistent with several previous reports. A retrospective cohort study reported a moderate association between elevated lipase levels (≥ 600 U/L) and radiologic severity indices such as the CTSI, supporting the concept that lipase may reflect local pancreatic injury rather than systemic disease severity [8].

Consistent with our findings, several studies have shown that established clinical scoring systems, particularly APACHE II and BISAP, are reliable predictors of mortality in acute pancreatitis [17–19]. In contrast, the prognostic value of the Glasgow–Imrie score remains controversial, with some studies reporting limited or no association with mortality, findings that are consistent with the results of the present analysis [20].

Among the evaluated prognostic scoring systems, Ranson, APACHE II, and BISAP were significantly associated with in-hospital mortality in univariate analyses. Importantly, in multivariable logistic regression analysis, the Ranson score remained independently associated with in-hospital mortality, whereas log-transformed serum lipase did not. This finding highlights the greater prognostic relevance of composite clinical scoring systems compared with isolated laboratory markers in predicting short-term outcomes.

An important and clinically relevant finding of this study was the lack of association between higher serum lipase levels and increased mortality. Several factors may explain this observation. First, elevated lipase levels may reflect early and localized pancreatic injury rather than sustained systemic inflammation. Second, the timing of serum lipase measurement relative to symptom onset may influence its apparent prognostic value, as enzyme levels typically peak early and decline even in the presence of clinical deterioration. Third, mortality in acute pancreatitis is more closely related to systemic complications such as persistent organ failure, sepsis, and metabolic disturbances, which are not adequately captured by serum lipase levels alone [21]. These considerations highlight the multifactorial nature of disease progression in acute pancreatitis and help explain why serum lipase does not reliably predict mortality when used in isolation.

After adjustment for the Ranson score, serum lipase was not independently associated with mortality, suggesting that it does not provide additional prognostic value beyond established clinical scoring systems. Although statistically significant, the low AUC value suggests that serum lipase alone has limited clinical utility for distinguishing higher-risk patients and should primarily be considered a supportive parameter rather than a definitive prognostic tool.

### Study limitations and future directions

This study has several limitations that should be acknowledged. First, its retrospective design may have introduced information bias related to incomplete or inconsistent documentation. Second, the single-center design may limit the generalizability of the findings to broader populations. Third, although a multivariable logistic regression model was used, the relatively small number of mortality events limited the number of variables that could be included without increasing the risk of model overfitting and may have reduced the stability of mortality-related estimates. In addition, the use of a predefined dichotomous cutoff for serum lipase may have reduced the predictive resolution of this inherently continuous biomarker.

Future prospective, multicenter studies with larger sample sizes, higher event rates, and standardized timing of biomarker measurements are needed to better clarify the relationship between serum lipase levels, clinical risk stratification, and patient outcomes.

## Conclusion

Serum lipase levels, using a cutoff value of 600 U/L, showed limited ability to identify higher-risk patients as reflected by the Ranson score (AUC: 0.585; sensitivity: 70.2%; specificity: 57.3%) and were not significantly associated with in-hospital mortality. In contrast, established clinical scoring systems, including Ranson, APACHE II, and BISAP, were significantly associated with mortality, underscoring their continued relevance in the prognostic assessment of acute pancreatitis.

The lack of association between elevated serum lipase levels and mortality highlights the complex pathophysiology of acute pancreatitis, in which pancreatic enzyme elevations do not necessarily reflect the systemic inflammatory response and organ dysfunction that drive fatal outcomes. These findings suggest that serum lipase should be considered a supportive marker rather than a standalone prognostic tool.

Future prospective, multicenter studies are needed to validate these findings and further clarify the role of serum lipase in the prognostic assessment of acute pancreatitis. Overall, our findings do not support any change in current prognostic assessment strategies and reinforce the limited standalone prognostic value of serum lipase.

## Data Availability

The datasets used and/or analyzed during the current study are available from the corresponding author on reasonable request.
